# Curious Sanitary Discoveries

**Published:** 1881-12

**Authors:** 


### CURIOUS SANITARY DISCOVERIES.

A municipal laboratory was not long since opened to the public in Paris, and many of its discoveries have been as curious and unexpected as they promise to be useful. For a small fee anybody can have analyzed samples of food, drinks, or anything connected with public hygiene. Among the articles in which adulteration was chiefly detected were wine, butter, and milk. The adulteration of wine consists principally of its being colored with fuchsine, and in many cases it does not contain a particle of the juice of the grape. Milk is deprived of its cream, and abundantly watered, and butter is frequently composed of anything but the ingredients of milk. Several samples of butter were found to be made with oil or suet, which were easily separated, and shown to the visitors, who accompanied the Prefect of Police to witness the experiments that were being performed in the laboratory. A sample that was exhibited as cream, which appeared as natural as possible, and of excellent flavor, was declared to have been manufactured with the residue of some red dye, mixed up with oil and sulphuric acid, in certain proportions. Some children's toys were examined, and those painted with red and blue were almost always found to contain poisonous substances, consisting principally of lead and copper. Even articles of perfumery were examined, which brought to light the most extraordinary examples of the skill brought into requisition for the accomplishment of fraudulent practices, for in several samples of scents of the most exquisite perfume, they were found to be manufactured with other materials than the essential oils of the flowers which they were intended to represent. But the most important discovery made was the presence in the nipples of some feeding bottles of a mass of vegetation, of cryptogamic nature, which, according to Dr. Faurel, bears a great analogy to the aphthous condition of the mouth frequently found in infancy, and he has induced the Academy of Medicine to investigate the matter, as he believes the condition referred to to be the origin of the intestinal affections, and particularly that form called "athrepsia," to which infants brought up by the bottle are subject.—*Med. and Surg. Rep.*

